# Microstructure, ion adsorption and magnetic behavior of mesoporous γ-Fe_2_O_3_ ferrite nanoparticles

**DOI:** 10.1039/d3ra01663c

**Published:** 2023-08-23

**Authors:** Farzad Nasirpouri, Sohiela Fallah, Ghader Ahmadpour, Elnaz Moslehifard, Aleksei Yu. Samardak, Vadim Yu. Samardak, Alexey V. Ognev, Alexander S. Samardak

**Affiliations:** a Faculty of Materials Engineering, Sahand University of Technology Tabriz Iran nasirpouri@sut.ac.ir f_nasirpouri@yahoo.com; b Institute of High Technologies and Advanced Materials, Far Eastern Federal University 10 Ajax bay, Russky Island Vladivostok 690922 Russia; c Faculty of Dentistry, Tabriz University of Medical Sciences Tabriz Iran; d Sakhalin State University Yuzhno-Sakhalinsk 693000 Russia

## Abstract

Magnetic nanoparticles with capacity for surface functionalisation have potential applications in water purification and biomedicine. Here, a simple co-precipitation technique was used to synthesize mesoporous ferrite nanoparticles in the presence of cetyltrimethylammonium bromide (CTAB) micellular surfactant. The as-synthesized ferrite nanoparticles were calcined at 250 °C for 5, 10, 15, and 24 h to remove the surfactant and create a mesoporous structure. The prepared samples were characterised using a wide range of analytical techniques. Microscopical images showed that all uncalcined particles have cauliflower shape without porosity. However, after calcination, surface and deep pores were created on the synthesized nanoparticles. In addition, transmission electron microscope (TEM) images of calcined nanoparticles revealed a wormhole-like structure, which is typical for the mesoporous architectures. Based on X-ray diffraction (XRD), the uncalcined and calcined samples exhibit pure Fe_3_O_4_ (magnetite) and γ-Fe_2_O_3_ (maghemite) ferrite phases, respectively. The γ-Fe_2_O_3_ nanoparticles demonstrated a high Brunauer–Emmett–Teller (BET) surface area with pore diameters smaller than 10 nm and a type IV isotherm similar to the mesopores. Hysteresis loops measured by vibrating sample magnetometry (VSM) showed the superparamagnetic nature for mesoporous γ-Fe_2_O_3_ nanoparticles. The first-order reversal curve (FORC) diagram revealed the formation of a mesoporous structure in calcined materials which reduces coercive distribution (*H*_c_) and magnetostatic interaction (*H*_u_) once compared to non-calcined samples. Mesoporous γ-Fe_2_O_3_ nanoparticles were successfully employed as an adsorbent for the removal of heavy metal ions of Pb(ii) from an aqueous solution. The highest lead ion adsorption was observed in mesoporous γ-Fe_2_O_3_ nanoparticles prepared with 3% CTAB.

## Abstract 2

Mesoporous γ-Fe_2_O_3_ ferrite nanoparticles prepared in the presence of cetyltrimethylammonium bromide micellular templates.

## Graphical abstract

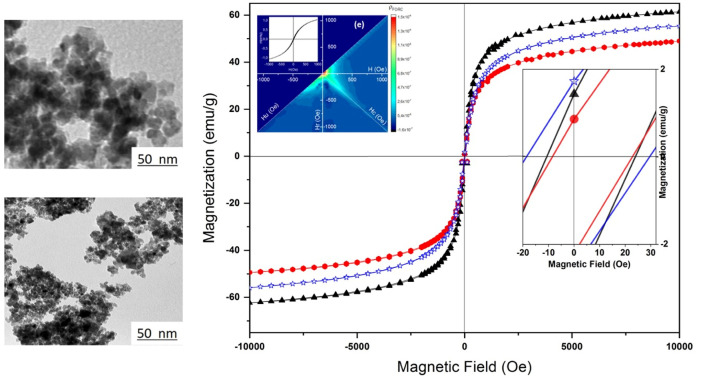

## Introduction

Porous materials can be categorized into three types based on pore size: macroporous (pores > 50 nm), mesoporous (pores 2–50 nm), and microporous (pores < 2 nm). Mesoporous nanoparticles have emerged as a fascinating class of materials with unique properties and diverse applications. These nanoparticles exhibit a well-defined porous structure with pore sizes ranging from 2 to 50 nm, offering a high surface area and large pore volume.^1^ Mesoporous nanoparticles, including mesoporous silica nanoparticles (MSNs),^2^ mesoporous carbon nanoparticles (MCNs),^3^ mesoporous metal oxide nanoparticles,^4,5^ mesoporous polymer nanoparticles,^6^ and mesoporous metal–organic frameworks (MOFs),^7^ are a versatile class of materials with well-defined mesoporous structures. These nanoparticles possess high surface area, adjustable pore sizes, and unique properties, rendering them highly suitable for a wide range of application.^8,9^

The synthesis of mesoporous materials involves two common methods: soft template and hard template. Soft template synthesis relies on the self-assembly of surfactant like cetyltrimethylammonium bromide (CTAB), followed by surfactant extraction. The hard template method utilizes pre-existing mesoporous structures and involves self-assembly within the limited space of the template. Soft template synthesis often yields materials with low crystallinity and thermal stability. Techniques like true liquid crystal templating (TLCT) and evaporation-induced self-assembly (EISA) are employed for ordered mesoporous materials.^10–12^ The TLCT method employs liquid crystal phases as templates for mesoporous structure formation, utilizing the self-organization of anisotropic fluids with long-range order. The precursor solution is introduced into the liquid crystal phase, which solidifies and is then removed, leaving behind mesoporous structures.^13,14^ In contrast, the EISA method involves controlled solvent evaporation. The precursor and a structure-directing agent, such as a surfactant or block copolymer, are dissolved in the solvent. As the solvent evaporates, the structure-directing agent self-assembles, resulting in mesophase formation. Solidification and template removal lead to mesoporous nanoparticles.^15,16^ Both methods provide effective routes for creating well-defined mesoporous materials.

Typical porous materials with high surface areas, like MSNs and MCNs, are widely used as adsorbents and catalysts in industry.^17^ However, they can occasionally present issues for industrial applications due to their poor separation efficiency, high regeneration temperatures, and inappropriate hydrophilic/hydrophobic characteristics. In some applications, having a large surface area alone is insufficient.^18^ For instance, adsorbents must be highly capable of adsorption, sensitive to specific pollutants like radioactive particles, hazardous metal ions, organic and inorganic solutes/anions, recyclable with high separation efficiency, and simple to regenerate. In contrast, magnetic ceramics are a type of metal oxide that has many advantages, including high electrical resistivity, ease of synthesis, and resistance to corrosion and wear. Among magnetic ceramics, iron oxide that can exist in several phases such as magnetite (Fe_3_O_4_), hematite (α-Fe_2_O_3_), and maghemite (γ-Fe_2_O_3_), has been used in a variety of fields and applications.^19,20^ Two major classes of iron oxide, magnetite and maghemite, have fascinating magnetic properties that make them suitable candidates for a range of applications, including electromagnetic adsorbers, high-frequency switch modes, heavy ion adsorbents, magnetic recording devices, and, in particular, biomedical ones.^21^ Maghemite (γ-Fe_2_O_3_) is a recyclable soft magnetic material that can be easily separated using a magnetic separation method.

The most common method for synthesizing mesoporous maghemite nanoparticles involves oxidizing synthesized magnetite nanoparticles in the presence of CTAB. Initially, magnetite (Fe_3_O_4_) nanoparticles are synthesized using a wet chemical procedure in the presence of CTAB as a surfactant.^22,23^ The CTAB molecules adsorb onto the surface of the magnetite nanoparticles, stabilizing their growth and preventing agglomeration. Subsequently, the surfactant is removed from the Fe_3_O_4_ nanoparticles through an oxidation process. This oxidation step transforms the magnetite into maghemite (γ-Fe_2_O_3_) while creating a mesoporous structure within the nanoparticles.

Mesoporous maghemite nanoparticles has a lot of potential applications: drug delivery, cancer cell destruction (magnetic hyperthermia), enhanced MRI imaging, catalysis, energy storage.^24,25^ Also, mesoporous maghemite nanoparticles are effective in removing various heavy metals from aqueous environments. For instance, in order to successfully remove cadmium (Cd^2+^) ions from contaminated water medium, a mesoporous magnetic composite, γ-Fe_2_O_3_-functionalized cross-linked chitosan (γ-Fe_2_O_3_@CS), was utilized.^26^ Also, γ-Fe_2_O_3_ nanoparticles with polyrhodanine coating were used to remove heavy metal ions (Hg(ii)) from aqueous solution.^20,27^ By utilizing their unique properties, mesoporous maghemite nanoparticles offer a promising solution for the remediation of heavy metal-contaminated water, contributing to the improvement of water quality and environmental health.^26,27^

In this comprehensive study, we conducted an in-depth investigation into the synthesis, microstructural characterization, magnetic properties, and adsorption efficiency of lead ions (Pb^2+^) from aqueous solutions using mesoporous maghemite nanoparticles. Our research aimed to explore the potential applications of these nanoparticles in water treatment and environmental remediation. The nanoparticles were synthesized through the oxidation of magnetite, which was prepared using the co-precipitation method with varying concentrations of CTAB surfactant. By examining different CTAB content, we aimed to assess the impact of surfactant content on the properties of the resulting mesoporous maghemite nanoparticles.

## Experimental

### Materials

The chemicals used for the synthesis of superparamagnetic mesoporous magnetite include divalent iron chloride (FeCl_2_), trivalent iron chloride (FeCl_3_·6H_2_O), cetyltrimethylammonium bromide (CTAB), sodium hydroxide (NaOH), ethanol (C_2_H_2_OH), deionized water (H_2_O), and liquid nitrogen with a purity of 99.9% supplied from Merck and Sigma-Aldrich.

### Synthesis

The method of Srivastava *et al.*^28^ was adopted for the synthesis of superparamagnetic mesoporous ferrite powder. Due to the different precursors of the process, Reaction (1) was considered.^29^

1 FeCl_2_ + 2FeCl_3_·6H_2_O + 8NaOH ⇒ Fe_3_O_4_ + 8NaCl + 10H_2_O

First, a solution of metal salts was obtained by dissolving appropriate amounts of iron chlorides in ethanol and deionized water. Then, a separate solution of CTAB with different contents (0, 1.5, 3, 5, 20 and 30%) was prepared in 10 mL of ethanol. The two solutions were mixed followed by adding 60 mL of deionized water. A NaOH solution (2 M) was prepared in ethanol at 80 °C and dropwise added to CTAB and iron chloride solution until the pH of the suspension reached 11. The color of the suspension altered from yellowish brown to blackish brown upon adding NaOH, reflecting the reaction and the formation of iron oxide. The resulting precipitates were separated by a centrifuge and washed several times with deionized water and ethanol. The sediments were then placed in a freeze dryer to decline the temperature below 0 using liquid nitrogen. The iron oxide precipitates were dried in a freeze dryer for 6 h at −40 °C and under a vacuum of 450 Torr. They were heat-treated at 250 °C for 5, 10, 15, and 24 h in the furnace. The calcination process was only carried out to remove CTAB from the synthesized structure and form a mesoporous structure in iron oxide nanoparticles. Table 1 displays the codes for samples of ferrite nanoparticles synthesized before and after calcination according to various CTAB percentages.

**Table 1:** Sample number/codes of ferrite nanoparticles prepared before and after calcination with various CTAB content

% of CTAB	Uncalcined samples	Calcined samples 250 °C
0	S1	C1
1.5	S2	C2
3	S3	C3
5	S4	C4
20	S5	C5
30	S6	C6

### Characterisation

Field emission scanning electron microscope (FESEM) (model SEM 950, Carl Zeiss) equipped with an energy dispersive X-ray (EDS) spectrometer and transmission electron microscope (TEM) (model Hitachi H-7000) were used to investigate the surface structure, morphology, size and size distribution of synthesized nanoparticles, as well as the effect of surfactant (CTAB) and calcination time on the size and size distribution of nanoparticles. Crystallographic structure of synthesised samples determined by X-ray diffraction using a Bruker, Germany, model D8 ADVANCE with a copper tube, a voltage of 40 kV, and a maximum current of 40 mA. The XRD pattern of the samples was taken between the angles of 20 to 65°. The detector scanned the sample with a step size of 0.01° every 4 seconds. Fourier transform infrared spectroscopy (FTIR) was used to identify the bonds in the synthesized mesoporous nanoparticles using a TENSOR 27 model made in Germany by Brucker Company. The analysis of N_2_ adsorption–desorption isotherms was used to check the porosity on the surface of the samples. The model of nitrogen gas adsorption and desorption device was BELSORP MINI II made by BEL company in Japan. The magnetic properties of the synthesized samples were measured using a vibrating sample magnetometer (VSM) manufactured by Magnetic Daneshpajoh Company in Kashan, Iran. The FORC measurements were carried out using a LakeShore VSM 7410 magnetometer. A standard solution proposed by Madhu Kumari and co-workers were used for using synthesized mesoporous superparamagnetic γ-Fe_2_O_3_ nanoparticles in lead adsorption.

## Results and discussions

### Morphology

**Fig. 1:**
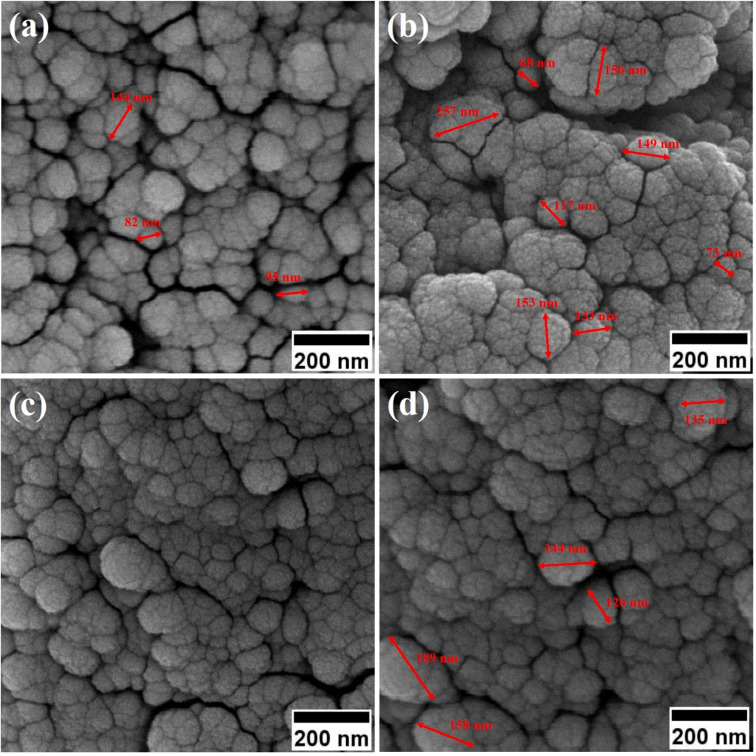
FESEM image of synthesized ferrite nanoparticles before calcination with CTAB of (a) 1.5%, (b) 5%, (c) 20% and (d) 30%.

**Fig. 2:**
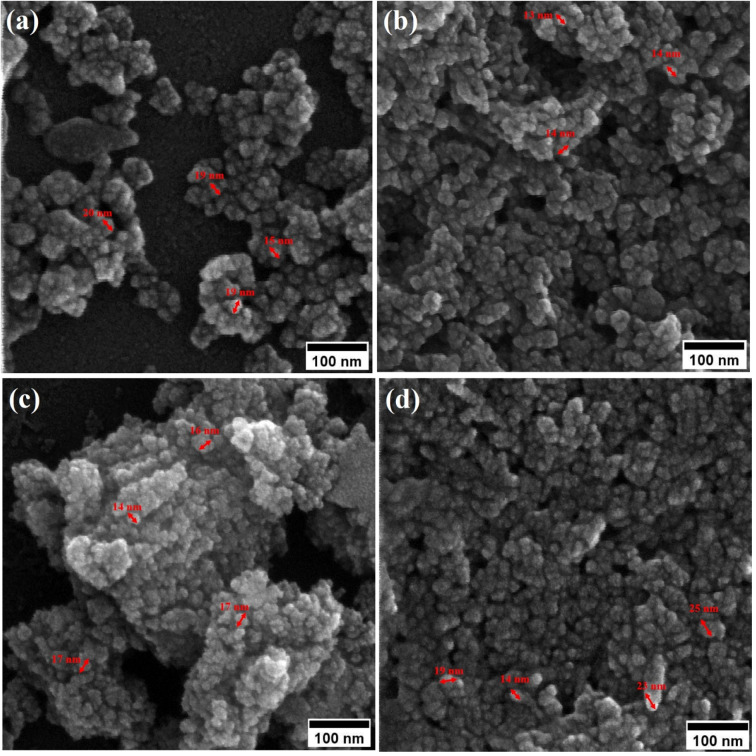
FESEM image of synthesized ferrite nanoparticles after calcination with CTAB of (a) 1.5%, (b) 5%, (c) 20% and (d) 30%.

Field-emission scanning electron microscopy (FESEM) was used to assess the morphology of superparamagnetic mesoporous iron oxide nanoparticles. Fig. 1 and 2 show FESEM images of nanoparticles synthesized before calcination (S2, S4, S5, and S6 samples) and after 15 h of calcination (C2, C4, C5, and C6 samples) at 250 °C, respectively. As can be seen, uncalcined samples included bulk particles (Fig. 1), which can be assigned to the presence of the surface activators in the synthesized samples before calcination as the size of the same sample was shrunk after calcination and no cauliflower accumulation can be observed after calcination (Fig. 2). The images of the nanoparticles before calcination were recorded at 100k× magnification due to their adhesion to each other and their larger size; while the images of the particles after calcination (Fig. 2) were taken at the magnification of 330k× due to their smaller microstructure.^30^

Table 2 lists the mean particle size of the samples based on the FESEM images in Fig. 1 and 2.

**Table 2:** Comparison of the mean particle size synthesized with different amounts of the surface activator (CTAB) before (samples of group S) and after calcination (samples of group C)

No	wt% of CTAB	Mean particle size before calcination (nm)	Mean particle size after calcination (nm)
1	0	—	40
2	1.5	129	18
3	5	138	14
4	20	150	17
5	30	152	21

By enhancing the CTAB content in the samples (before calcination), their size was enlarged. The mean size of the uncalcined particles was initially 129 nm, which was enhanced to 152 by increasing the CTAB content. The nanoparticles had similar structures before calcination with no porosity as the pores were blocked by CTAB. The calcination is aimed to remove CTAB from the structure of the nanoparticles to form surface and depth porosity on/inside the superparamagnetic mesoporous nanoparticles. A comparison of the calcined nanoparticles indicated that the rise in the CTAB content first decremented the mean size of the nanoparticles followed by an increase. The same behavior was also observed in mesoporous iron oxide materials synthesized using the soft-templating method by increasing the CTAB content, the concentration of the structure-directing agent is elevated. This can lead to a higher coverage of CTAB molecules around the growing nanoparticles and result in larger particle sizes. The excess CTAB molecules form a thicker layer around the particles, promoting their growth and reducing the surface energy, which contributes to the enlargement of the nanoparticles.^31^

**Fig. 3:**
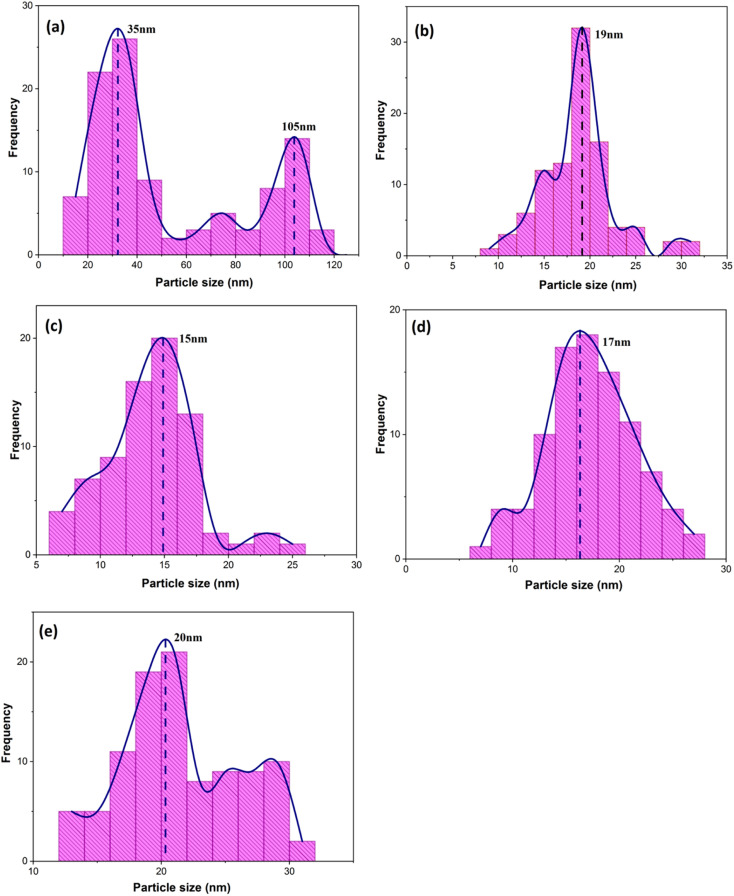
Size distribution diagrams of synthesized particles in the presence of various concentrations of CTAB: (a) without the presence of CTAB and (b) 1.5%, (c) 5%, (d) 20%, and (e) 30% of CTAB (extracted from Fig. 2 FESEM images).

**Fig. 4:**
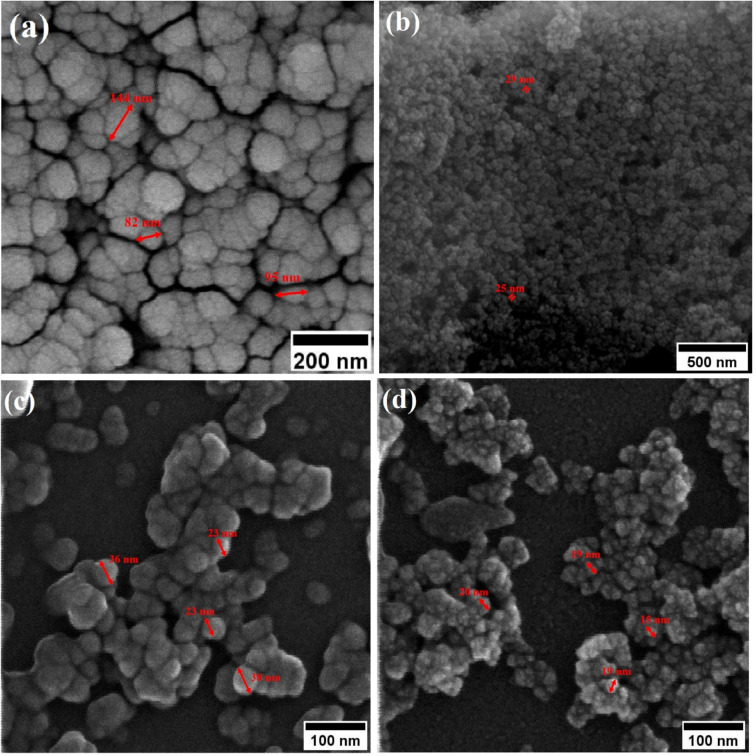
Morphology of iron oxide nanoparticles synthesized with 1.5% of CTAB (a) before calcination and after calcination for (b) 5 h, (c) 10 h and (d) 15 h at 250 °C.

**Fig. 5:**
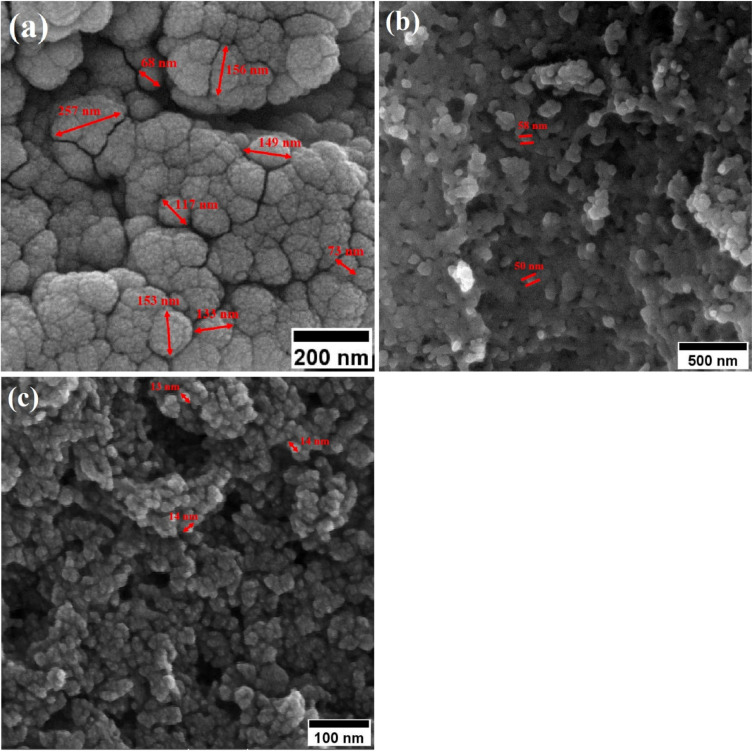
Morphology of iron oxide nanoparticles synthesized with 5 wt% of CTAB (a) before calcination and after calcination for (b) 5 h, and (c) 15 h at 250 °C.

When CTAB is added to a solution, it forms micelles due to its lyotropic (liquid crystal) nature. The size of these micelles can be influenced by the concentration of CTAB in the solution. At low CTAB content, the micelles formed are relatively small in size. As the CTAB content increases, the size of the micelles initially decreases due to the increased surfactant-to-solvent ratio. This behavior is often observed in the regime of low to moderate CTAB content. However, as the CTAB content continues to increase, a critical point is reached where the micelles start to grow in size. This can be attributed to the increased availability of CTAB molecules, which leads to the formation of larger micelles. At this point, the surfactant-to-solvent ratio becomes more favorable for the growth of larger micelles, resulting in an increase in their size. Regarding the complete removal of surface activators, such as CTAB, from structures with higher CTAB content, it can indeed pose challenges. When a higher amount of CTAB is used, it can become more difficult to remove all the surfactant molecules during subsequent purification steps, such as calcination or washing processes. Incomplete removal of CTAB can lead to residual surfactant molecules remaining on the surface of the nanoparticles.^32–34^ These residual surfactants can act as adhesive agents, causing the particles to adhere to each other. This phenomenon can result in particle aggregation or agglomeration, affecting the overall structure and properties of the mesoporous iron oxide nanoparticles.^35^ The size distribution of the synthesized nanoparticles was calculated by FESEM analysis of Fig. 2 as depicted in Fig. 3. The size distribution of the particles synthesized in the absence of CTAB is also depicted in Fig. 3(a) (sample C1). In normal Gaussian diagrams, the highest frequency shows the mean size of the particles. In this regard, the nanoparticles synthesized without CTAB had a mean particle size of 35 and 105 nm. The size distribution of these particles also showed a wide diagram extending from 10 to 120 nm. The mean size of nanoparticles prepared in the presence of 1.5 wt% CTAB (sample C2) was 19 nm with a narrow distribution from 10 to 30 nm, while 90% of the particles fell below the size of 20 nm (Fig. 3(b)). A rise in the CTAB content from 1.5 to 5% (sample C4) led to narrower size distribution as the size distribution of particles varied from 5 to 25 nm, where 90% of the particles were smaller than 20 nm and larger than 5 nm (Fig. 3(c)). The mean size of these particles was estimated to be 15 nm. A rise in the CTAB content from 5 to 20% (sample C5) and 30% (sample C6) enhanced the size of the particles from 15 to 17 and 20 nm, respectively. The size distribution diagrams indicated that the size of particles, prepared in the presence of 20% CTAB, varied from 5 to 20 nm (Fig. 3(d)), which is similar to the particles synthesized with 5% of CTAB; however, 90% of these particles were smaller than 18 nm. The distribution of the particles synthesized by 30% of CTAB (sample C6) was wider (ranging from 11 to 30 nm) than those prepared with 5 and 20% of CTAB (sample C5). The samples prepared with various CTAB contents were calcined at 250 °C for 5, 10, 15, and 20 h to assess the influence of the calcination time on the morphology. Fig. 4 shows the morphology of iron oxide nanoparticles prepared with 1.5% of CTAB before (sample S2) and after calcination (sample C2) for 5, 10, and 15 h. As expected, a rise in the calcination temperature decremented the particle size and accumulation. The size of the bulk particles before the calcination process was 129 nm which declined to 27, 23, and finally 18 nm after calcination for 5, 10, and 15 h, respectively. The same trend can be observed in the case of iron oxide nanoparticles prepared with 5% of CTAB (samples S4 and C4) (Fig. 5). A comparison of the morphology of samples S4 and C4 in Fig. 5 revealed a decline in the size of the accumulated particles as well as nanoparticles. For sample S4 (Fig. 5(a)), the size of the accumulated particles was 138 nm which declined to 53 and 14 nm after 5 and 15 h of calcination, respectively (Fig. 5(b) and (c)). Based on FESEM images, more CTAB will be removed by prolonging the calcination time, leading to the formation of mesoscale pores. On the other hand, as agglomeration declined with the removal of the surface activators, the true size of the particles can be measured at high accuracy.

**Fig. 6:**
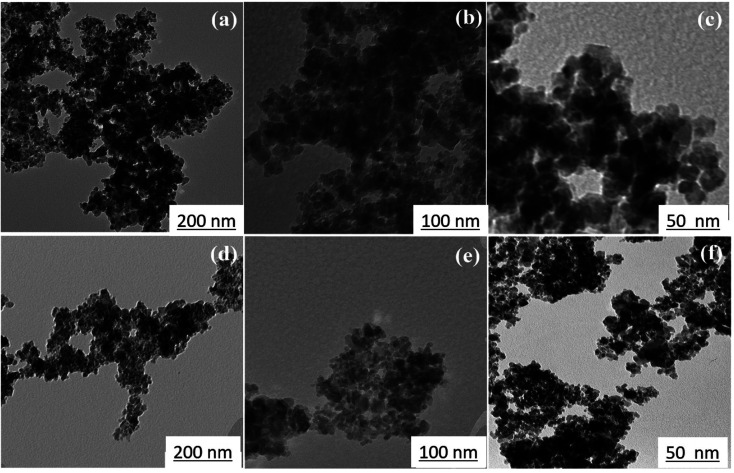
TEM images of mesoporous iron oxide nanoparticles prepared in the presence of (a, b and c) 1.5 wt% CTAB (sample C2), (d, e and f) 3 wt% CTAB (sample C3) after 24 h of calcination at 250 °C.

Fig. 6 shows TEM images at various magnifications of iron oxide nanoparticles calcined at 250 °C for 24 h with 1.5 wt% (sample C2) and 3 wt% (sample C3) CTAB. The samples contain nanoparticles with almost uniform size in the range of 10–20 nm, as shown in Fig. 6(a) and (d). The uniform and tubular structure of the samples generated, with worm-like mesopores, is also clearly visible in Fig. 6(b) and (d). Mesoporous iron oxide nanoparticles are partially agglomerated spherical particles, as shown in Fig. 6(c) and (f). The presence of agglomerated particles can be attributed to the growth mechanism of particles during the CTAB-assisted co-precipitation synthesis of mesoporous iron oxide nanoparticles. In this technique, CTAB surfactant plays a crucial role as a soft template for the formation of the nanoparticles. During the co-precipitation process, the Ostwald ripening mechanism becomes predominant in the growth of the nanoparticles. Ostwald ripening occurs due to the difference in surface free energy between smaller and larger nanoparticles. Smaller nanoparticles possess higher surface free energy compared to larger nanoparticles. As the reaction progresses, nanoparticles of various sizes are present in the solution. The higher surface free energy of smaller nanoparticles drives the diffusion of material from smaller to larger particles. This phenomenon is known as Ostwald ripening or particle coarsening. The material from smaller nanoparticles tends to dissolve and redeposit on the surfaces of larger nanoparticles, leading to their growth at the expense of smaller particles. However, during this growth process, there can be instances where the aggregation or agglomeration of particles occurs. Aggregation refers to the formation of clusters or aggregates of particles, whereas agglomeration refers to the sticking together of individual particles. The agglomeration/agglomeration of particles can be attributed to various factors, including the presence of residual surfactant, the concentration and distribution of particles in the solution, and the conditions during the co-precipitation process. To minimize agglomeration/agglomeration and promote the formation of well-dispersed mesoporous iron oxide nanoparticles, careful optimization of the synthesis parameters, such as the concentration of CTAB, reaction temperature, and reaction time, is required. Additionally, subsequent purification steps, such as washing and calcination, play a crucial role in removing residual surfactants and stabilizing the nanoparticles, ensuring their uniform dispersion and preventing further agglomeration/agglomeration. It can also be seen from Fig. 6(c) and (f) that the particle size of sample C3 is smaller than that of sample C2, which can be related to the more amount of CTAB used in the sample C3. A greater amount of CTAB causes more gas to be released from the burning of CTAB during the calcination process, increasing porosity and decreasing particle size.^36^ It should be noted, however, that a higher concentration of CTAB may have an adverse effect on the morphology of mesoporous nanoparticles. In this case, CTAB may not be completely removed from the nanoparticle structure during the calcination process, resulting in a decrease in porosity and an increase in particle size.

**Fig. 7:**
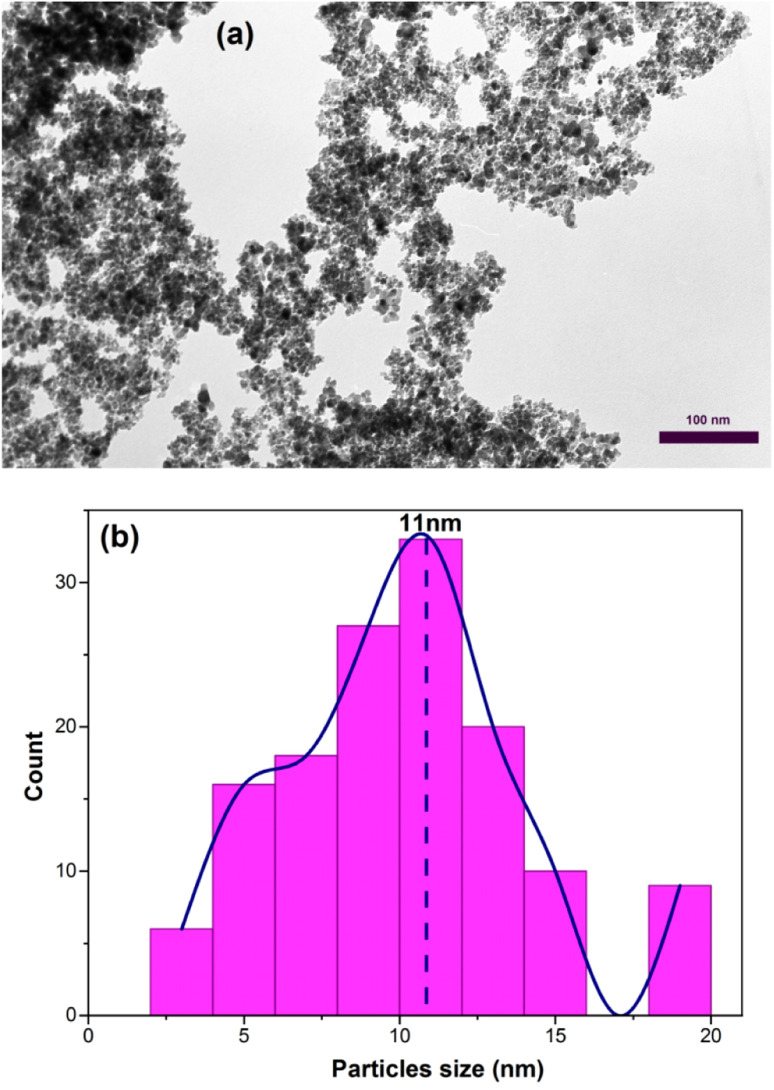
(a) TEM image of mesoporous iron oxide nanoparticles prepared by using 3% of CTAB after calcination for 15 h (b) size distribution diagram of the same sample.

Fig. 7(a) shows the transmission electron microscopy (TEM) image of the sample C3 after 15 h of calcination. As depicted in Fig. 7(a), the nanoparticles have a wormhole-like structure, which is the characteristic of mesoporous structures. This structure is a defining characteristic of mesoporous materials and contributes to their high surface area and pore volume. The term “wormhole-like” refers to the presence of a network of interconnected pores within the nanoparticles, resembling tunnels or channels. These pores have a well-defined size and shape, typically in the mesoscale range (2 to 50 nanometers in diameter). The interconnected nature of these pores creates a three-dimensional framework, providing a large surface area for interactions with molecules and facilitating the diffusion of substances within the nanoparticle. The wormhole-like structure in mesoporous nanoparticles is typically formed during the synthesis process. In the case of mesoporous iron oxide nanoparticles, the addition of CTAB surfactant during co-precipitation or other synthesis methods plays a crucial role in creating and stabilizing this structure. CTAB molecules self-assemble around the nanoparticles, forming a template that guides the growth and arrangement of the mesoporous structure. The resulting wormhole-like structure in mesoporous iron oxide nanoparticles offers several advantages for various applications. The high surface area and pore volume enable efficient adsorption and diffusion of molecules, making them desirable for applications such as catalysis, drug delivery, and sensing. Additionally, the interconnected pores provide a means for controlled release of encapsulated substances or targeted transport within the nanoparticles. The size distribution of the particles was also assessed by selecting 150 particles as showed in Fig. 7(b). The particles exhibited an ultra-narrow size distribution (3–15 nm) with high overlap.^37^

### Phase and elemental analysis

**Fig. 8:**
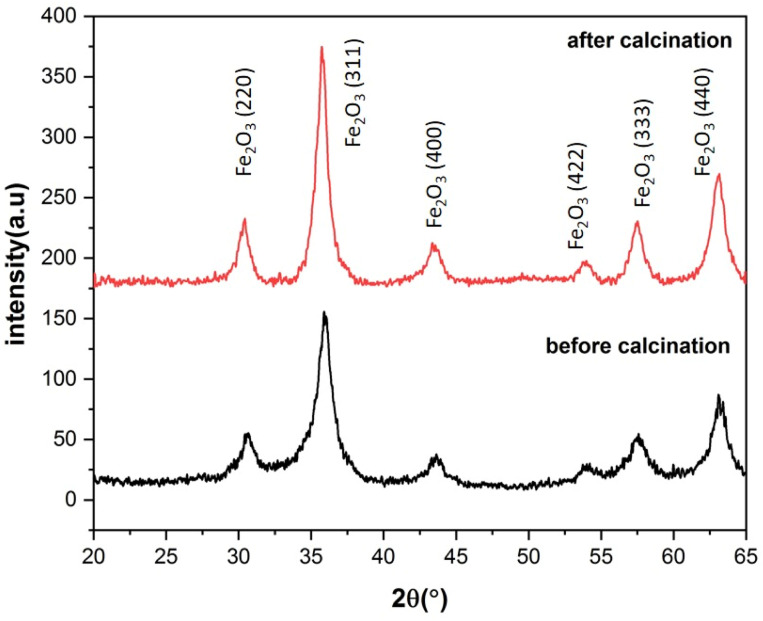
X-ray diffraction patterns of ferrite nanoparticles synthesized with 3% of CTAB before and after calcination for 24 h at 250 °C.

The XRD patterns of the S3 and C3 (calcinated for 24 h) samples are displayed in Fig. 8. The XRD pattern of sample S3, with a prominent peak (311) at about 35.9° and planes (220), (400), (422), (511), and (440) at about 30.5, 43.7, 53.8, 57.3, and 62.9° matches well with the Fe_3_O_4_ (magnetite) phase according to JCPDS code: 96-900-5817. There was no evidence of other iron oxide phases in sample S3. Sample C3 shows similar planes with a little shift in peak locations. This shows that, while the lattice constant differs between the two samples, the crystal structure (cubic spinel lattice) is identical. Furthermore, the intensity of the peaks is increased after calcination at 250  C, which indicates an improvement in the crystallinity and removal of the surfactants from the nanoparticle pores. The evaluation of the data in the High Score Plus software package suggests the presence of a maghemite phase (γ-Fe_2_O_3_) in sample C3 with JCPDS code: 96-900-6317. The most common method of synthesizing γ-Fe_2_O_3_ is to oxidize Fe_3_O_4_ in air at temperatures around 200–300 °C. Therefore, it can be inferred that the Fe_3_O_4_ phase entirely transforms into the γ-Fe_2_O_3_ phase following the calcination of sample S3 at 250.  C^38^ The lattice constant decreases from 8.471 to 8.351 Å as magnetite transforms into maghemite. The decreasing lattice constant could be explained by the ionic radii of the Fe^2+^ and Fe^3+^ cations. Since Fe^2+^ cations have a larger ionic radius, the Fe–O distance is also larger. Because of the oxidation of Fe^2+^ cations to Fe^3+^ cations during the conversion of maghemite to magnetite, the spacing reduces and leads to a decrease in the lattice constant. The crystallite size of the particles synthesized was calculated by the Debye–Scherer equation:2
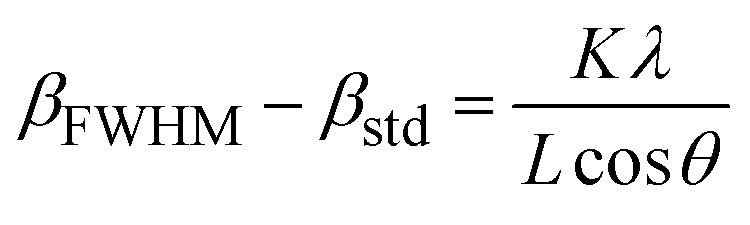
in which *β*_FWHM_ shows the peak broadening at half the maximum intensity. *β*_std_ also indicates the peak broadening due to the instrument error, often considered as 0.02. *K* denotes the shape factor of the crystallite in the range of 0.62 to 2.08, which was taken 0.98 for spherical crystallites with cubic symmetry. *L* and *λ* respectively represent the crystallite size and wavelength of the X-ray produced by the copper cathode (1.5406 Å). *θ* also shows half of the diffraction angle. Based on eqn (2), the crystallite size of C3 and S3 samples was determined to be 10.5 and 9.5 nm, respectively. These calculations showed close consistency with the mean size of the particles defined from the size distribution diagram of nanoparticles of sample C3 (calcinated for 15 h), in which most of the particles had the size of 11 nm (Fig. 8(b)). The crystallite size is smaller or equal to the particle size as particles may be sometimes formed by the combination of several crystallites, leading to a polycrystalline structure. For nanometric particles, crystallite size could be equal to the particle size. A comparison of the most abundant particles prepared by 3 wt% of CTAB (size = 11 nm) with the Debye–Scherer-determined crystallite size (10.5 nm) indicated that the particle size is almost equal to the crystallite size of γ-Fe_2_O_3_.^39,40^ We also comment on the effect of the micelles on the formation of this crystalline phase structure. The phase transition from Fe_3_O_4_ (magnetite) to γ-Fe_2_O_3_ (maghemite) may occur in iron oxide nanoparticles, including mesoporous ones, under certain conditions. This phase transition is influenced by various factors such as temperature, time, and the surrounding environment. The presence or absence of the CTAB (cetyltrimethylammonium bromide) micellular template does not directly dictate whether the phase transition will occur. CTAB is often used as a surfactant or template in the synthesis of mesoporous materials, including iron oxide nanoparticles. It can help control the size, shape, and mesoporous structure of the nanoparticles. However, the phase transition from Fe_3_O_4_ to γ-Fe_2_O_3_ can also occur in the absence of a CTAB template, depending on the synthesis conditions and subsequent treatment of the nanoparticles. The phase transition is typically induced by thermal treatment, such as calcination, at elevated temperatures. During the heating process, Fe_3_O_4_ nanoparticles can transform into γ-Fe_2_O_3_ due to the redistribution of iron atoms and oxygen vacancies.^41^ This phase transition is often accompanied by changes in the crystal structure, magnetic properties, and surface chemistry of the nanoparticles. It is important to note that the specific conditions required for the phase transition can vary depending on the synthesis method, precursor materials, and thermal treatment parameters. Therefore, while the CTAB template can influence the synthesis and morphology of mesoporous iron oxide nanoparticles, the phase transition from Fe_3_O_4_ to γ-Fe_2_O_3_ can occur in the presence or absence of the CTAB template under appropriate synthesis and thermal treatment conditions.

**Fig. 9:**
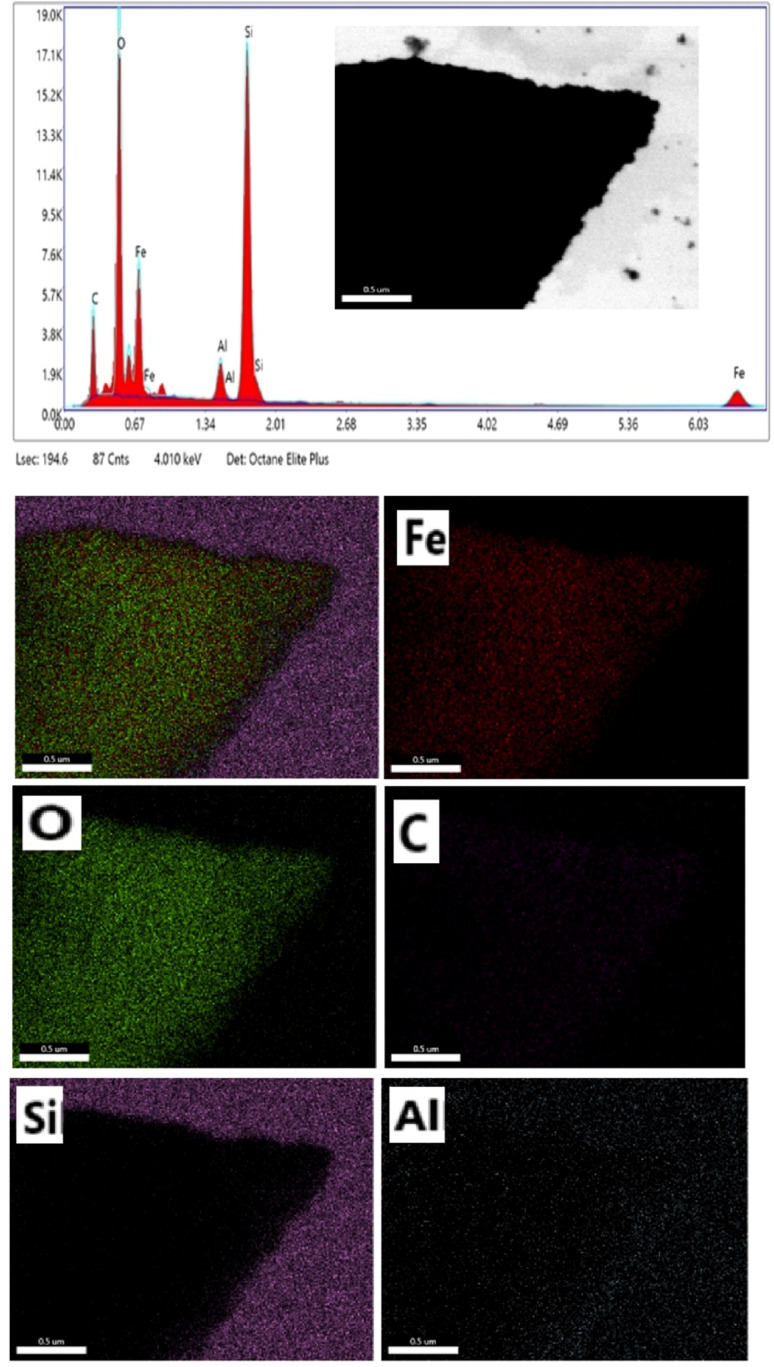
EDS and elemental mapping of mesoporous γ-Fe_2_O_3_ nanoparticles prepared in the presence of 3% of CTAB (sample C3).

EDS analysis was used to determine the composition of the mesoporous γ-Fe_2_O_3_ nanoparticles for 5% CTAB after 24 h of calcination at 250 °C, as shown in Fig. 9. According to EDS analysis, the product consists of Fe, O, C, Si and Al elements. The presence of carbon in the iron oxide nanoparticle composition indicates that CTAB is not completely eliminated during the calcination process, whereas the Si and Al peaks in the EDS spectra are related to the semiconductor substrate on which the sample is placed. The iron-containing region in the EDS spectra of iron oxide nanoparticles was between 0–1 and 6–7 keV, while the oxygen-containing region was between 0–1 keV. The peaks around 0.7, and 6.5 keV were connected with Fe binding energies, while the peak at 0.5 keV was related with oxygen binding energies. As a result, the EDS spectra for sample C3 confirmed the formation of γ-Fe_2_O_3_ nanoparticles.^42^ In order to better understand the distribution of the constituent elements of mesoporous γ-Fe_2_O_3_ nanoparticles, the EDS mapping analysis of sample C3 is shown in Fig. 9. Elemental mapping analysis confirms the uniform distribution of Fe and O elements in the mesoporous γ-Fe_2_O_3_ nanoparticles, as well as the residual carbon traces from CTAB, which are uniformly spread throughout the sample. For the full removal of CTAB from iron oxide composition, a high calcination temperature (500  C) is required.^43^ But increasing the calcination temperature will lead the growth of particles and the loss of the mesoporous structure. Therefore, the presence of carbon in the final product is normal due to the low calcination temperature.

### FTIR spectroscopy

**Fig. 10:**
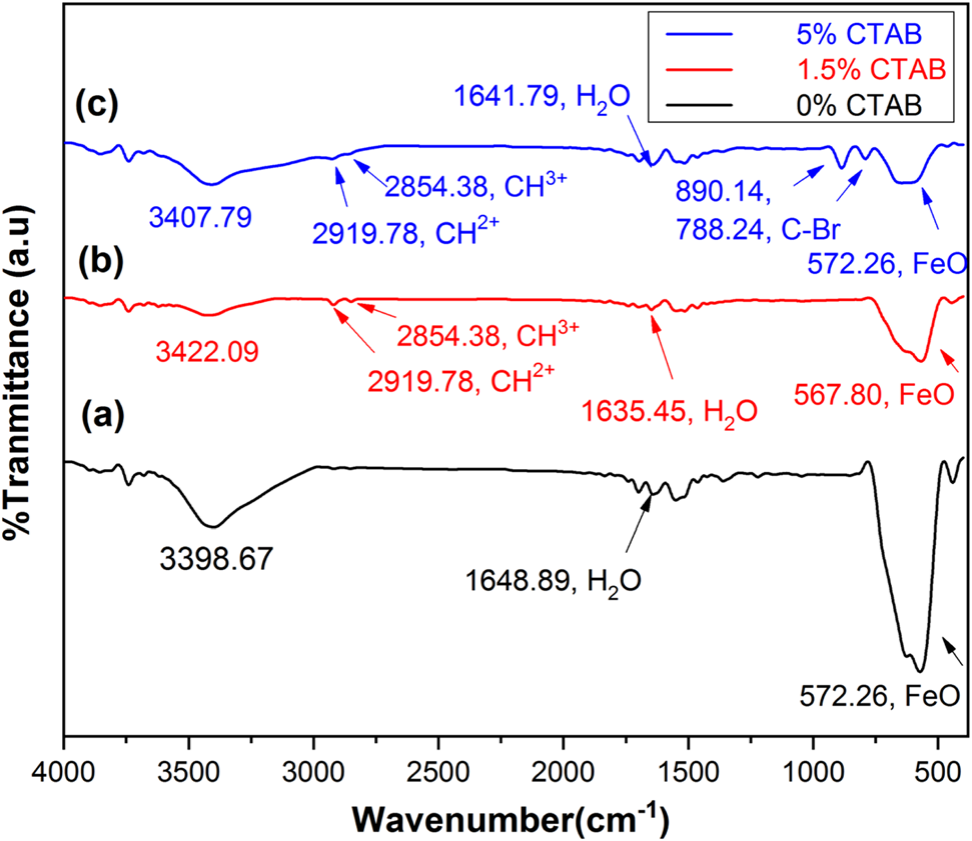
FTIR spectra of synthesized mesoporous γ-Fe_2_O_3_ nanoparticles in the presence of (a) 0%, (b) 1.5% and (c) 5% of CTAB after 5 h of calcination at 250 °C.

Fourier transform infrared spectroscopy (FTIR) was utilized to assess the chemical bonds on the surface of the samples. Fig. 10 shows the FTIR results of the mesoporous γ-Fe_2_O_3_ nanoparticles for C1, C2, and C4 after 5 h of calcination at 250 °C. The peaks (low frequency bands) between the wavenumber of 567 cm^−1^ and 577 cm^−1^ correspond to the stretching vibrations of the Fe–O bond, which are consistent with those observed in the spinel maghemite (γ-Fe_2_O_3_) phase.^44^ The peaks emerging at 1635–1648 cm^−1^ in all three samples can be attributed to water and ammonia molecules. The peaks at 3390–3420 cm^−1^ are related to the decline of the water content in the samples after calcination. The peaks at 2919.78 and 2854.38 cm^−1^ in the samples of C2 and C4 can be ascribed to the hydrocarbon chains (CH^3+^ and CH^2+^) in the CTAB. The presence of an adsorption peak related to the C–Br bond at 788.24 and 890 cm^−1^ in the C4 sample suggests the incomplete removal of the surface activator during the calcination process.^45,46^

### Nitrogen adsorption–desorption isotherms of superparamagnetic mesoporous γ-Fe_2_O_3_ nanoparticles

**Fig. 11:**
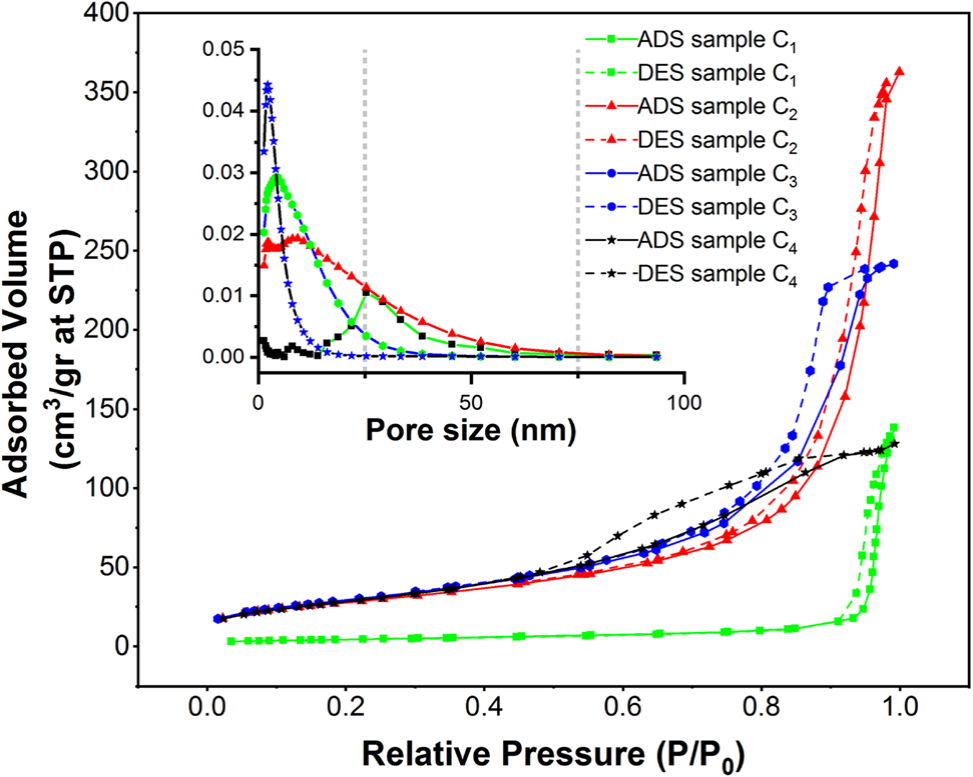
Nitrogen gas adsorption and desorption isotherms (BET) of synthesized mesoporous γ-Fe_2_O_3_ nanoparticles in the presence of 0, 1.5, 3, and 5% of CTAB calcined at 250 for 24 h, as well as the pore size distribution diagram for the same samples using BJH theory.

In order to assess specific surface area, the nitrogen adsorption and desorption test was performed on four samples of mesoporous γ-Fe_2_O_3_ nanoparticles that had been calcined for 24 h at 250 °C. These nanoparticles were similar except for their CTAB content which was different. The nitrogen adsorption–desorption isotherms (BET), as well as the size distribution of the pores determined by BJH calculations, are presented in Fig. 11, for C1, C2, C3 and C4 samples and the important information of these diagrams is also listed in Tables 3 and 4. As suggested in Fig. 11, all the isotherms are of IV type with H_1_ hysteresis which is typical of the mesoporous materials. This implies that the surface porosity is in the range of 2–50 nm.^47^ The size distribution of the pores was estimated by the BJH method as depicted in the left corner of Fig. 11.

**Table 3:** The specific surface area of mesoporous γ-Fe_2_O_3_ nanoparticles prepared with various concentrations of CTAB using BJH theory

Sample	% of CTAB	Calcination time (h)	Specific surface area (m^2^ g^−1^)	Average pore size (nm)
C1	0	24	19.2	—
C2	1.5	24	121.2	8
C3	3	24	141.8	2
C4	5	24	138	4

**Table 4:** The specific surface area of mesoporous γ-Fe_2_O_3_ nanoparticles manufactured utilizing various theories, as well as particle size determination using BET surface area

Sample	% of CTAB	Specific surface area BJH (m^2^ g^−1^)	Specific surface area Langmuir (m^2^ g^−1^)	Specific surface area BET (m^2^ g^−1^)	Specific surface area *t*-plot (m^2^ g^−1^)	Particle size estimation *D*_EBT_ (nm)
C2	1.5	121.2	124.9	110.5	95.1	11
C3	3	141.8	140	125.9	95.9	9.7
C4	5	138.1	134	120	92.6	10

Based on the results, the porosity size distribution of the C2, C3 and C4 samples was about 8, 2, and 4 nm. The porosity size distribution of the C3 sample prepared in the presence of 3% CTAB was very narrow and comparable to the value (2.1–2.5 nm) reported by Mitra.^31^ But the distribution of pore size was wider in the C4 sample prepared with 5% CTAB; this width was even higher for the C2 sample synthesized by 1.5% CTAB. This can decrement the specific surface area of these samples.

As depicted in Table 3, a rise in CTAB content from 1.5 to 3% enhanced the specific surface area to 141.88 m^2^ g^−1^ while the pore size declined from 8 to 2 nm. Further enhancement in the CTAB percentage to 5% slightly decremented the specific surface area to 138 m^2^ g^−1^ and the pore size also showed an increase to 4 nm compared to the sample prepared with 1.5% of CTAB. The specific surface area obtained in this study was significantly greater than the value (7.9 m^2^ g^−1^) reported by Zhang and co-workers.^48^ Assuming spherical particles with uniform size distribution (which is not far from reality in this research), eqn (3) can be employed to estimate the particle size regarding the specific surface area determined by the BET method.3
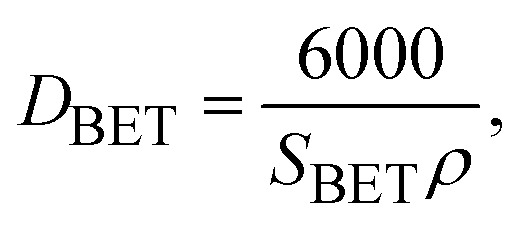
in which *D* shows the particle diameter in nanometer; while *ρ* (g cm^−3^) and *S*_BET_ (m^2^ g^−1^) respectively represent the density and specific surface area of γ-Fe_2_O_3_ nanoparticles.^49^ By substituting *S*_BET_ from Table 3 into eqn (3), the size of nanoparticles can be obtained as listed in Table 4. A comparison of the BET-determined size of the nanoparticles with SEM and TEM images indicated that the BET-estimated size of the nanoparticles in the presence of all three amounts of surfactant were lower than the particle size determined by SEM and TEM images.

Adsorption isotherms are mathematical models used to describe the relationship between the amount of adsorbate (gas or solute) adsorbed on an adsorbent surface and the equilibrium pressure or concentration of the adsorbate. There are several types of adsorption isotherms, including: Langmuir, BET (Brunauer–Emmett–Teller), BJH (Barrett–Joyner–Halenda) and *t*-plot isotherms. The Langmuir isotherm assumes monolayer adsorption on a homogeneous surface with a limited number of identical adsorption sites. It assumes that adsorption is reversible and that there is no interaction between adsorbed molecules. The Langmuir equation is often used for systems where adsorption occurs on a surface with limited sites. The BET isotherm is specifically designed for the analysis of gas adsorption on solid surfaces with multilayer adsorption. It assumes the formation of a monolayer on the surface with subsequent layers exhibiting lower adsorption energies. The BET model is commonly used for mesoporous materials to determine surface area, pore size distribution, and adsorption capacity. In mesoporous nanoparticles, the BET isotherm is often used due to its ability to describe the multilayer adsorption behavior on porous surfaces. Mesoporous materials have a high surface area and well-defined pore structures, making the BET model suitable for characterizing their adsorption properties. The BET model allows for the determination of specific surface area, pore volume, and pore size distribution, which are important parameters in understanding the adsorption capacity and performance of mesoporous nanoparticles in various applications. Additionally, the BET model provides insights into the surface properties and accessibility of the adsorption sites in mesoporous materials, making it a valuable tool for optimizing the design and application of mesoporous nanoparticles in fields such as water treatment, environmental remediation, and separation processes. The BJH isotherm is used to analyse the pore size distribution in mesoporous materials. It is based on the desorption branch of the nitrogen adsorption isotherm, where the adsorbed nitrogen is gradually desorbed from the material at various relative pressures. By analysing the desorption data, the BJH model can provide information about the size distribution of the pores in the material, including the mesopores. The *t*-plot model is another method used to analyse the pore size distribution, specifically for microporous and mesoporous materials. It is based on the analysis of the adsorption branch of the nitrogen adsorption isotherm. The *t*-plot model calculates the thickness of the adsorbed nitrogen layer at different relative pressures and uses this information to estimate the pore size distribution. This model is particularly useful for materials with narrow pore size distributions. Table 4 compares the specific surface area determined by these various methods (BET, BJH, Langmuir, and *t*-plot).

### Magnetic properties of the synthesized nanoparticles

#### Hysteresis loop measurement

**Fig. 12:**
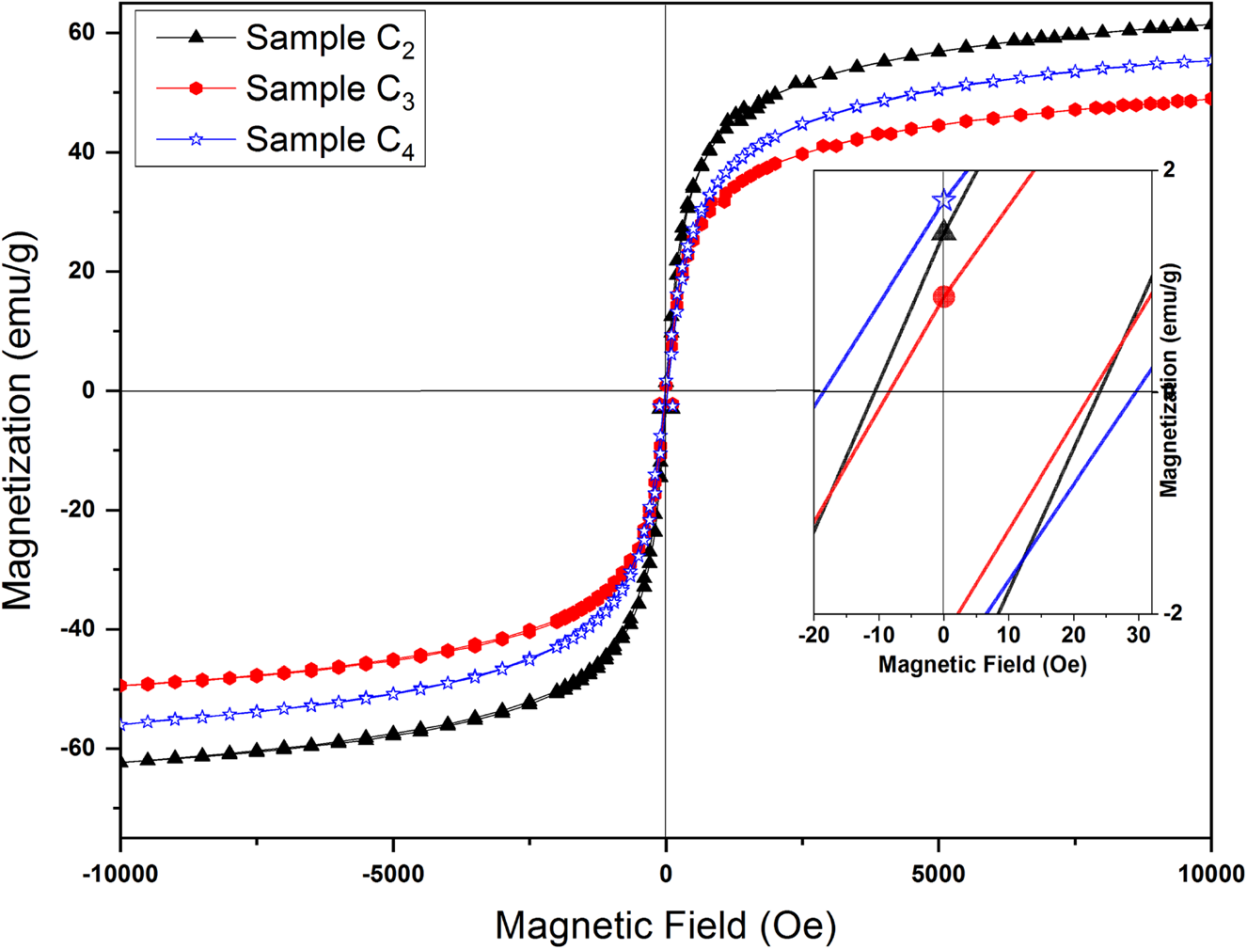
Magnetic hysteresis loops of samples synthesized in the presence of 1.5%, 3% and 5% CTAB calcined at 250 °C for 24 h. Magnified hysteresis loops are presented on the inset.

The magnetic properties of mesoporous γ-Fe_2_O_3_ nanoparticles were assessed by LakeShore VSM 7400. The samples of C2, C3 and C4 (calcinated for 24 h at 250 °C) were selected for measurements, their hysteresis loops are depicted in Fig. 12. All three samples exhibited S-shape hysteresis with close to zero remnant magnetization (*M*_r_) and coercivity (*H*_c_) in the range of 5–30 Oe (inset in Fig. 12). The linear paramagnetic incline can originate from the sample holder. Table 4 lists *H*_c_ along with saturation magnetization (*M*_s_). As widely shown in the literature, transition from single-domain to superparamagnetic state with decreasing of the volume of nanoparticle occurs in the point, defined as critical superparamagnetic diameter, for spherical γ-Fe_2_O_3_ nanoparticles it is about 15 nm for room temperature.^50^ Particle sizes in this work are 9–11 nm and 8–19 nm, obtained by BET method and SEM/TEM analysis respectively, and well within this range. This, with combination with S-shaped hysteresis loop, allows us to suppose that investigated nanoparticles can be of superparamagnetic nature. Non-zero values of coercive force can be assigned to the wide distribution of the size of synthesized particles (Fig. 3) as well as the incomplete removal of CTAB from the nanoparticle microstructure. Superparamagnetic materials have high saturation magnetization but zero coercive force and remanent magnetization, which provides the possibility of controlling their movement in an external magnetic field, without agglomeration under the influence of magnetostatic interactions.^51^ Therefore, they could find many applications in different fields, including the removal of heavy metals.

*H*_c_ and *M*_s_ values for samples prepared with different amount of CTAB are presented in Table 5. Maximum saturation magnetization at the external field of 10 kOe is 61.06 emu g^−1^ (C2 sample), which is smaller than the saturation magnetization of bulk γ-Fe_2_O_3_ (74 emu g^−1^).^52^ Such a low value compared to bulk samples can be assigned to the high surface-to-volume ratio of mesoporous nanoparticles. A large percentage of their atoms are located on the surface, which causes changes such as the formation of crystal lattice vacancies, breakage of chemical bonds, water adsorption on the particle surface, and redistribution of cations all of which lead to a decrease in the saturation magnetization. The surface effects also lead to the formation of a dead magnetic layer on the surface of the particles, which can ultimately reduce the saturation magnetization of nanoparticles compared to their bulk materials.^53^ As one can see from the Table 4, the saturation magnetization for C3 and C4 samples is low compared to C2 sample, which can be related to the decrease in particle size with the increase in CTAB amount.

**Table 5:** Coercivity and saturation magnetization of mesoporous γ-Fe_2_O_3_ nanoparticles with various CTAB percentages and particle sizes

Sample	CTAB%	Magnetization saturation *M*_s_ (emu g^−1^)	Coercivity *H*_c_ (Oe)	Particle size estimation *D*_EBT_ (nm)
C2	1.5	61.06	23.83	20 (from TEM image)
C3	3	49.06	22.78	11 (from TEM image)
C4	5	55.33	29.43	14 (from SEM image)

### First-order reversal curve (FORC) diagram method

Hysteresis loops can offer a general and direct insight into the magnetic behaviour of the sample. Advanced methods such as the first-order reversal curve (FORC) diagram method have been recently developed for an accurate and detailed understanding of the properties and magnetic behaviour of materials.^54^ In particular, FORC diagrams offer the possibility of evaluating the distribution of the coercive forces and magnetostatic interactions in the investigated sample.^55^ The FORC distribution can be calculated as the second-order complex derivative of the magnetization (*M*) in terms of the magnetic field (*H*) and the reversal field (*H*_r_) according to the following equation.^56^4
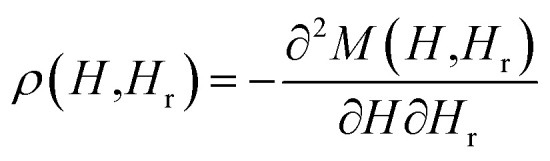
where *ρ* is FORC distribution density. In the two-dimensional FORC diagram, coercive fields (*H*_c_) and interaction fields (*H*_u_) are used instead of *H* and *H*_r_ axes for better readability of the chart. These axes rotated by 45° relative to the *H* and *H*_r_ (ref. 57) and are defined analytically as follows:5
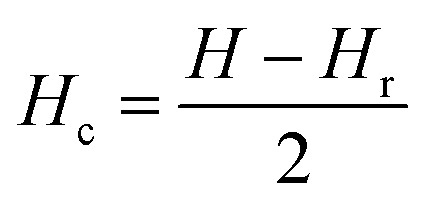
6
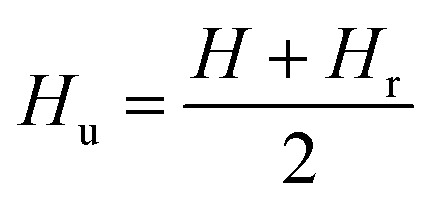


**Fig. 13:**
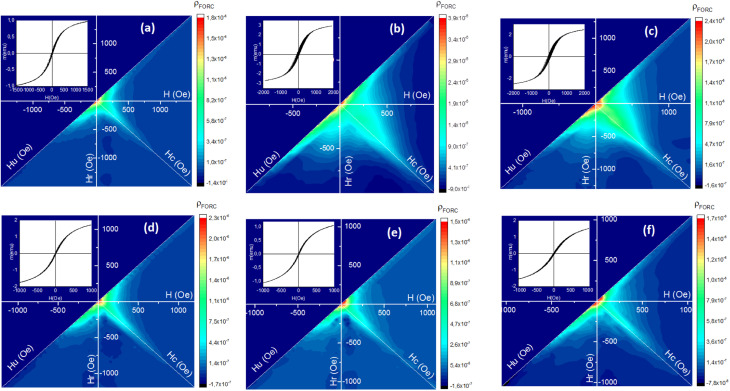
FORC diagrams of the ferrite nanoparticles prepared in the presence of 1.5% of CTAB (a and d), 3% of CTAB (b and e), and 5% of CTAB (c and f). Images of (a) to (c) for samples before calcination and (d) to (f) for samples after calcination (d, e, f).

Fig. 13 shows the FORC diagrams of the mesoporous γ-Fe_2_O_3_ nanoparticles prepared in the presence of 1.5, 3, and 5% CTAB before (S2, S3 and S4 samples) and after calcination (C2, C3 and C4 samples) for 24 h at 250 °C. Elongation (non-elongation) along the *H*_u_ axis could suggest the presence (absence) of magnetic interactions between the particles. The *H*_c_ shows the coercive force distribution of magnetic particles, and the elongation of the FORCs diagram along this axis can be the sign of the presence of nanoparticles with different coercive forces as result of large particle size distribution in the sample.

Since superparamagnetic particles have an almost zero coercive field, FORC-diagrams for them usually have main peak with highest *ρ* in the close to zero *H*_c_ region. The distribution of *ρ* on FORC-diagrams of all samples can be divided into two different regions - one is characterized by close to zero coercive forces and relatively wide distribution along the *H*_u_ axis, and the other represents elongated distribution along the *H*_c_ axis with almost no interactions.^58^ The intensities of these two regions are different before and after calcination, it also varies with CTAB content variations.

The ridge analysis of *ρ* of the FORC diagram showed higher broadening along the *H*_c_ axis in the uncalcined samples (Fig. 13(b) and (c)) when compared to the calcined ones (Fig. 13(d)–(f)). As one can see, maximum *H*^FORC^_c_ on the half-height for uncalcined samples can reach up to 500 Oe, a rather large coercive force characteristic for single-domain nanoparticles with bigger sizes. After the calcination *H*_c_ distribution shrinks notably, with decreasing of maximum *H*^FORC^_c_ to 100 Oe, signifying the domination of superparamagnetic phase. TEM and SEM results of the nanoparticles before calcination also showed wider size distribution of the particles compared to the calcined samples. The only exception is S2 sample (1.5% of CTAB) FORC-diagram which does not changes notably.

A significant elongation can be observed along the *H*_u_ axis for the uncalcined samples due to their large particle size and particle agglomeration because of the presence of CTAB. This indicates higher magnetostatic interactions in the uncalcined samples.^59^ After the calcination, the distance between the γ-Fe_2_O_3_ particles increases due to the post-calcination porosity which naturally decreases the magnetostatic interaction and results in shortening of *H*_u_ distribution on FORC-diagram for calcinated samples. Table 6 shows the average coercivity forces (*H*^FORC^_c_), interaction fields (*H*_u_), squareness ratio (*M*_r_/*M*_s_) obtained from FORC-diagrams.

**Table 6:** Coercivity and saturation magnetization of Fe_3_O_4_ and mesoporous γ-Fe_2_O_3_ nanoparticles with various CTAB percentages and particle sizes

Samples		CTAB%	*H* ^FORC^_c_ (Oe)	*H*_u_ (Oe)	*M*_r_/*M*_s_
Uncalcinated (Fe_3_O_4_)	S2	1.5	40	161	0.047
	S3	3	132	295	0.118
	S4	5	170	221	0.104
Calcinated (γ-Fe_2_O_3_)	C2	1.5	18	150	0.031
	C3	3	14	120	0.022
	C4	5	20	191	0.026

### Application of mesoporous magnetite nanoparticles as lead ion adsorbent

Heavy metals have been distributed in the environment as a result of human activities. People can be exposed to these metals by consuming food or contaminated water. The gradual accumulation of these metals in the body leads to adverse effects. Lead is one of the most dangerous heavy metals in food which can damage the respiratory and immune systems. This metal is highly toxic for children due to its destructive influence on their nervous systems. No organ of a child's body is immune to the effects of lead poisoning.^60–62^ In the present research, synthesized mesoporous γ-Fe_2_O_3_ nanoparticles were used to remove Pb^2+^ from an aqueous solution.

**Fig. 14:**
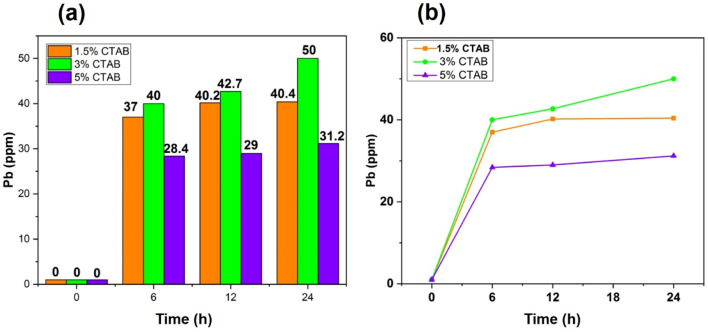
(a) The influence of CTAB% on lead adsorption efficiency in the synthesis of mesoporous γ-Fe_2_O_3_ nanoparticles (b) the influence of mesoporous γ-Fe_2_O_3_ nanoparticle contact duration on the percentage of lead adsorption.

To investigate the effect of the surface activator (CTAB) content used during the synthesis of superparamagnetic mesoporous γ-Fe_2_O_3_ nanoparticles on the lead adsorption efficiency, three C2, C3 and C4 (calcined for 24 h) were selected. The effect of contact time of synthesized nanoparticles with the lead ion-containing solution on the Pb^2+^ adsorption efficiency was explored by preparing three solutions of 100 mg of Pb^2+^ per litter (50 mL) at a pH of 5. For this purpose, 0.1 g of C2, C3 and C4 samples were added to each container containing lead ions and placing them on the shaker for 6, 12, and 24 h. After separating the superparamagnetic nanoparticles from the suspensions, the secondary concentration of lead ion was measured by atomic adsorption. Table 7 shows the initial and final concentration of lead ions in the suspensions containing nanoparticles of C2, C3 and C4 samples after 6, 12, and 24 h of contact with nanoparticles. The influence of the CTAB content on the adsorption capacity of nanoparticles is shown in Fig. 14(a). The general trend shows a rise in lead ion adsorption by enhancing the CTAB content due to the increase in the porosity of the synthesized nanoparticles. This trend can be seen in the case of nanoparticles of C2 and C3 samples with 1.5 and 3% of CTAB, respectively. But nanoparticles synthesized with 5% of CTAB (sample C4) exhibited the opposite behaviour as their lead ion adsorption declined despite an increase in CTAB percentage and possibly the increment in porosity. The reason could be improper CTAB removal from the pores of the synthesized particles during the calcination process. This sample needs longer calcination for improvement of the result.^63^

**Table 7:** Initial and final concentration of lead in the solution after 6, 12 and 24 h contact with synthesized nanoparticles in the presence of 1.5%, 3% and 5% of CTAB

Sample	CTAB%	Initial concentration of Pb in solution (mg L^−1^)	Pb concentration in solution after 6 h (mg L^−1^)	Pb concentration in solution after 12 h (mg L^−1^)	Pb concentration in solution after 24 h (mg L^−1^)
C2	1.5	100	63	59.8	60.6
C3	3	100	60	57.3	49.9
C4	5	100	71.6	71	68.8

The effect of the contact time of synthesized nanoparticles on the adsorption percentage of lead ions in the solution is depicted in Fig. 14(b). As can be seen, an increase in the contact time of nanoparticles with lead ions enhanced the percentage of adsorbed lead ions. The equilibrium adsorption capacity of lead ions can be calculated by eqn (7).^64^7
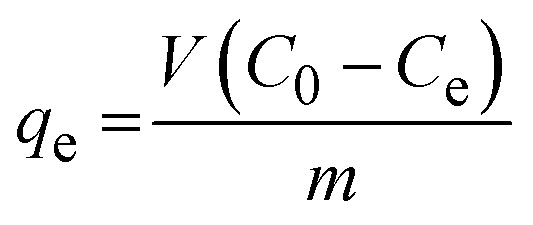


In which, *q*_e_ is the equilibrium adsorption capacity (mg g^−1^); *v* shows the volume of solution (L); *C*_0_ and *C*_e_ represent the initial and equilibrium concentration of lead ion in the solution (mg L^−1^), respectively; and *m* is the mass of synthesized nanoparticles. Equilibrium adsorption capacity was employed to compare the results of the present research with previous studies as listed in Table 8. According to Table 8, among the created superparamagnetic mesoporous γ-Fe_2_O_3_ nanoparticles in the current investigation, sample C2 has the highest equilibrium lead ion adsorption capacity, surpassing that of the Fe_3_O_4_ nanospheres made by Kumari *et al.*^65^

**Table 8:** Comparison of the equilibrium adsorption capacity of synthesized ferrite-based nanoparticles for the adsorption of heavy elements

No.	Nanoparticles used	Synthesis method	Equilibrium adsorption capacity of the mentioned element (mg g^−1^)	Ref
1	γ-Fe_2_O_3_	Using CTAB and co-precipitation method	Pb^2+^(1.5% CTAB): 20.7	This work
			Pb^2+^(3% CTAB): 25	
			Pb^2+^(3% CTAB): 15.6	
2	Fe_3_O_4_ nanospheres	One-step solvothermal method	Pb^2+^: between 6.64 and 8.90	65
3	ϒ-Fe_2_O_3_	Sol – Gel	Cr^4+^: 19.2	66
4	ϒ-Fe_2_O_3_	Sol – Gel	Cu^2+^: 26.8	67
5	Hematite	Co-precipitation	Cu^2+^: 84.46	68
6	Core (Fe_3_O_4_)-Shell (Al–Mn)	Hydrothermal and chemical self-assembly process	Cr^4+^: 150	69
7	Fe3O4@APS@AA-co-CA MNPs	Co-precipitation/free-radical polymerization	Zn^2+^: 43.4 – Cd^2+^: 29.6 – Pb^2^: 166.1	70
8	γ-Fe_2_O_3_	Wet chemical	Zn^2+^: 4.49 – Cd^2+^: 1.75 – Pb^2^: 10.55	71
9	γ-Fe_2_O_3_	Co-precipitation	Pb^2^: 25	72
10	γ-Fe_2_O_3_	Hydrothermal	Arsenic ions: 73.2	73

## Conclusions

Iron oxide nanoparticles based on spinel ferrite were synthesized by the co-precipitation method in the presence of different contents of CTAB as a micellular surfactant. The XRD patterns revealed that the synthesized samples were composed of the magnetite (Fe_3_O_4_) phase before to calcination, but following calcination at 250 °C, it was entirely converted into maghemite (γ-Fe_2_O_3_). FESEM and TEM images confirmed the formation of nanoparticles with the mesoporous structure after calcination process. To investigate the impact of calcination duration on the removal of surfactant and the formation of mesoporous γ-Fe_2_O_3_ structure and also to study its magnetic properties, Fe_3_O_4_ nanoparticles were calcined at constant temperature of 250 °C for 5, 10, 15 and 24 h. The obtained results showed that the CTAB content used in the synthesis of nanoparticles, as well as the calcination duration, had a significant effect on the particle size, particle size distribution, pore size, and specific surface area of the final nanoparticles. The optimal amount of surface activator was 3% as the sample prepared in the presence of 3% of CTAB showed the highest specific surface area (140 m^2^ g^−1^). Increasing the calcination time improved the mesoporous structure due to enhancing CTAB removal from the nanoparticle microstructure. The investigation of magnetic properties of the calcined samples showed an s-shaped hysteresis, with close to zero coercivity and remnant magnetization. In combination with sizes of the nanoparticles, it can be assumed that nanoparticles are in superparamagnetic state with good approximation. The non-zero values of coercive force can be assigned to the wide size distribution of the synthesized particles as well as the incomplete removal of CTAB from the nanoparticle microstructure, which act as pinning sites. The lowest coercive force (8 Oe) was related to mesoporous nanoparticles synthesized with 3% of CTAB, therefore, this sample had the best magnetic properties for the proposed usage. The FORC diagrams showed that uncalcined samples are characterized with high interaction fields and coercivity due to their large particle size and particle agglomeration because of the presence of CTAB. After the calcination process, the width of the coercivity force and interaction field decreased significantly due to the formation of a mesoporous structure and overall decrease of nanoparticles' sizes. Furthermore, mesoporous nanoparticles with 3% of CTAB exhibited the highest lead ion adsorption. Such a high adsorption capacity can be attributed to their mesoporous structure and large specific surface area.

## Author contributions

Farzad Nasirpouri: conceptualizing and initiating of the project, administration and supervision of the project, funding acquisition, validation of synthesis of nanoparticles, formal analysis of data, writing-original draft; Soheila Fallah: investigation, methodology of synthesis of nanoparticles, examinations and analysis (TEM, SEM, XRD, FTIR, BET, VSM), data; Ghader Ahmadpour: investigation, data curation, visualization (SEM, TEM, XRD, EDX, VSM, FORC diagram method), writing-original draft; Elnaz Moslehifard: investigation, data curation, writing-original draft; Aleksei Yu Samardak: investigation, data curation, visualization and writing (VSM, FORC diagram method); Vadim Yu Samardak: investigation, data curation, writing-original draft; Alexey V. Ognev: investigation, data curation, visualization (TEM); Alexander S. Samardak: validation, formal analysis, visualization, writing (magnetic properties, FORC-diagram analysis).

## Conflicts of interest

There are no conflicts to declare.

## Supplementary Material
